# Exosome-Mediated Transfer of X-Motif-Tagged Anti-MiR-33a-5p Antagomirs to the Medial Cells of Transduced Rabbit Carotid Arteries

**DOI:** 10.3390/biology13120965

**Published:** 2024-11-24

**Authors:** Goren Saenz-Pipaon, Bradley K. Wacker, Lianxiang Bi, Alexis Stamatikos, David A. Dichek

**Affiliations:** 1 Department of Medicine, Division of Cardiology, University of Washington, Seattle, WA 98195, USA; bwacker@uw.edu (B.K.W.); lbi@cardiology.washington.edu (L.B.); ddichek@uw.edu (D.A.D.); 2 Department of Food, Nutrition, and Packaging Sciences, Clemson University, Clemson, SC 29634, USA; adstama@clemson.edu

**Keywords:** antagomir, X-motif, exosome, MiR-33a-5p, ABCA1, vascular, gene therapy, HDAd

## Abstract

Atherosclerosis is caused by the accumulation of cholesterol within intimal smooth muscle cells (SMCs) and macrophages. However, the transporter ATP-binding cassette subfamily A, member 1 (ABCA1), can remove cholesterol from these intimal, cells reducing atherosclerosis. Antagomir-mediated inhibition of miR-33a-5p, a microRNA that represses ABCA1 translation, promotes ABCA1-dependent cholesterol efflux and may impede atherosclerosis development. In our previous work, transducing cultured endothelial cells (ECs) with a helper-dependent adenoviral vector (HDAd) that expresses X-motif-tagged anti-miR-33a-5p enhanced antagomir packaging into EC-derived exosomes, which delivered the antagomir to cultured SMCs and macrophages. In this present study, we tested whether in vivo transduction of rabbit carotid artery endothelium can deliver an X-motif-tagged anti-miR-33a-5p to subendothelial cells. Rabbit carotid endothelial cells were transduced in vivo with an HDAd expressing anti-miR-33a-5p either with or without the X-motif (*n* = 11 arteries per vector). Contralateral carotids received HDAd that express scrambled oligonucleotides. Three days after transduction, the antagomir—without the X-motif—was detected in the intima but not in the media of transduced carotids (*p* = 0.062). The X-motif antagomir was detected in 82% of the intimal extracts (9 out of 11 carotids) and 27% of medial samples (3 out of 11 carotids, *p* = 0.031). However, the X-motif did not significantly enhance antagomir delivery to the media (*p* = 0.214 vs. non-X-motif antagomir). Expression of the antagomirs—with and without the X-motif—was sub-stoichiometric in ECs and SMCs. No antagomir-related changes in miR-33a-5p or ABCA1 expressions were detected. Despite its potential as a therapeutic strategy, our exosome-targeted gene transfer system requires further improvements to enhance antagomir expression and delivery to the subendothelial cells.

## Simple Summary

Atherosclerosis, the leading cause of heart disease, involves cholesterol buildup within the artery wall. Current treatments are ineffective in removing excess cholesterol from the diseased arteries. Accordingly, increasing expression of cholesterol export proteins such as ATP-binding cassette subfamily A, member 1 (ABCA1), may enhance cholesterol efflux and impede atherosclerosis development. We previously showed that endothelial cells (ECs) transduced with a viral vector can release small delivery vehicles (exosomes) loaded with a therapeutic molecule (antagomir) targeting miR-33a-5p, which inhibits ABCA1. The addition of a short ‘X-motif’ sequence enhanced antagomir packaging into exosomes and delivery into artery wall cell types in vitro. In the present study, we explored whether this EC-based exosome-mediated strategy can deliver the anti-miR-33a-5p into the medial cells of the rabbit artery in vivo. The X-motif antagomir was found in the medial cells of the transduced arteries; however, this strategy still requires further refinement to enhance antagomir expression and delivery within the artery wall. In future studies, this strategy could help reduce plaque buildup in arteries, offering a new avenue for atherosclerosis treatment.

## 1. Introduction

Cardiovascular diseases, primarily of atherosclerotic origin, remain the leading cause of mortality worldwide [1]. Medications aimed at lowering low-density lipoprotein cholesterol (LDL-C) such as statins or inhibitors of proprotein convertase subtilisin/kexin type 9 (PCSK9) reduce acute cardiovascular events [2,3]. However, these types of current treatments are only partially effective, leaving substantial residual risk even after intense LDL-C reduction [3,4]. Plaque size is also minimally affected by current LDL-C-lowering treatments [5]. In contrast, strategies that remove cholesterol from the artery wall could decrease plaque size and improve plaque stability. This is supported by the negative correlation between plasma cholesterol efflux capacity and coronary artery disease, which is a stronger association than with plasma LDL-C [6]. Accordingly, therapies aimed to enhance reverse cholesterol transport (RCT)—the physiological mechanisms that remove cholesterol from arteries—could potentiate the therapeutic effect of LDL-C lowering.

The cholesterol acceptor apolipoprotein AI (ApoAI) plays a pivotal role in RCT by participating in the formation of nascent high-density lipoprotein (HDL) particles that aid in shuttling cholesterol out of arteries [7,8]. Experimental evidence shows that systemic injections of ApoAI or HDL promote lipid removal from the artery wall and decrease atherosclerosis in preclinical animal models and clinical trials [9,10,11]. However, other human trials testing HDL-raising drugs [e.g., niacin or cholesterol ester transfer protein (CETP) inhibitors], ApoAI-mimetic peptides, or reconstituted complexes of ApoAI have not yet proven any appreciable clinical benefit [12,13]. These types of therapies are also considered life-long, with associated costs and the risk of possible systemic side effects.

As an alternative approach to the aforementioned therapies, our group employed vascular wall-targeted ApoAI gene therapy using helper-dependent adenoviral (HDAd) vectors [14,15,16]. With our strategy, lipid-poor ApoAI is directly produced by the transduced artery endothelium, adjacent to the lipid-rich plaque, and can provide durable transgenic expression for at least 48 weeks. This approach impeded atherosclerosis progression and enhanced regression of small-to-medium-sized atherosclerotic lesions in rabbits [14,15,16]. However, our HDAd-mediated gene transfer system did not stimulate the regression of larger atherosclerotic lesions in the carotid arteries of rabbits [15]. This limitation might be explained—at least in part—by a reduction in cholesterol efflux proteins such as ATP-binding cassette subfamily A1 (ABCA1) in advanced atherosclerotic lesions [17,18]. Indeed, ABCA1 is shown to decrease within lipid-laden intimal smooth muscle cells (SMCs) [17]. Because ApoAI-mediated cholesterol efflux depends on ABCA1 expression and functional ABCA1 is also needed for nascent HDL generation [19,20], upregulating ABCA1 levels within intimal cells—SMCs and macrophages—may potentiate the efficacy of ApoAI gene therapy.

ABCA1 is post-transcriptionally regulated by microRNAs (miRNAs) that repress its translation. MiR-33a-5p is a major post-transcriptional repressor of ABCA1, and thus, its inhibition de-represses ABCA1 and increases cholesterol efflux [21,22,23,24]. MiR-33a-5p is upregulated in human atherosclerotic lesions and exerts its pro-atherogenic functions via inhibition of genes involved in (i) cholesterol efflux (e.g., ABCA1); (ii) hydrolysis of lipid droplets (e.g., ATG5 and ATG12); and (iii) fatty acid oxidation (e.g., HADHB and CROT) [23,24,25]. MiR-33a-5p ablation de-represses ABCA1 and promotes cholesterol efflux in macrophages, which impedes atherosclerosis progression in mice [26,27]. However, systemic inhibition of miR-33a-5p also de-represses genes involved in fatty acid metabolism, thus causing adverse effects such as hepatic steatosis and hypertriglyceridemia [28,29]. Thus, vascular wall-targeted therapies that locally inhibit miR-33a-5p may enhance ABCA1 expression directly in the artery in which it is needed for atheroprotection while minimizing potential side effects.

Inhibiting miR-33a-5p in the vascular wall requires specialized delivery vehicles that can transfer anti-miR-33a-5p antagomirs into the subendothelial cells. In this regard, extracellular vesicles (EVs) serve as an endogenous cell-to-cell communication system that allows the transfer of miRNA, and other small RNAs, from endothelial cells (ECs) to SMCs. Indeed, EV-mediated miRNA transfer has demonstrated therapeutic promise both in vitro and in vivo [30,31,32]. We recently showed that EC-derived small EVs, or exosomes, could transfer anti-miR-33a-5p antagomirs to SMCs and macrophages in vitro [33]. Moreover, the incorporation of an X-motif sequence to this antagomir enhanced exosome-mediated transfer to both cultured SMCs and macrophages. This approach de-repressed ABCA1 and promoted ApoAI-mediated cholesterol efflux [33]. In our current study, we test whether anti-miR-33a-5p can be effectively expressed in vivo and examine whether incorporating the X-motif sequence enhances antagomir delivery to the subendothelial cells via EC-derived exosomes.

## 2. Materials and Methods

### 2.1. Animal Studies

Specific-pathogen-free New Zealand white rabbits (*n* = 11 males and *n* = 11 females; 3.0–3.5 kg; Western Oregon Rabbit Company, Philomath, OR, USA) were fed a normal rabbit chow (Purina Laboratory Rabbit Diet High Fiber, #5326, Gray Summit, MO, USA). After 1-week acclimatization to the animal facility, rabbits underwent neck surgery, during which viral vectors were intraluminally infused into both common carotid arteries, as described below. Rabbits were euthanized 3 days after vector infusion by intravenous injection of a pentobarbital/phenytoin euthanasia cocktail with confirmation by respiratory arrest and absence of a heartbeat. All animal studies were approved by the University of Washington Office of Animal Welfare.

We utilized rabbits because they present several advantages over rodents: (i) rabbits are medium-sized animals, and thus, they bridge the gap between mice and large animal models (e.g., pigs); (ii) they provide a larger amount of tissue, allowing us to perform more analyses with fewer animals; and (iii) wild-type rabbits fed a high-fat diet develop atherosclerotic lesions that resemble those of humans without requiring genetic mutations in genes such as APOE or LDLR, which is ideal for future studies testing our therapeutic approach [34].

### 2.2. Adenoviral Vectors

We utilized four distinct HDAd vectors from our previous study [33]: (i) HDAdAntimiR (expresses an antagomir that targets rabbit miR-33a-5p); (ii) HDAdScr (expresses a scrambled antagomir); (iii) HDAdXMoAntimiR (expresses the anti-miR-33a-5p antagomir tagged with an X-motif); and (iv) HDAdXMoScr (expresses the scrambled antagomir tagged with an X-motif). All the nucleotide sequences and schematic representations of the expression cassettes are available in Table S1 and Figure S1. The expression of all the transgenes is driven by the H1 promoter. Vector stocks were propagated in human embryonic kidney (HEK) 293Cre4 cells (Microbix Biosystems, Toronto, ON, Canada) and purified as described [35]. Concentrations of vector stocks (measured by spectrophotometry) were 1–4 × 10^12^ viral particles (vp)/mL. Real-time quantitative polymerase chain reaction (qPCR) revealed that E1A-containing genomes were <1 in 10^6^ total vector genomes and helper virus contamination was <1% of total vector genomes [36]. The qPCR primer and probe sequences for the detection of E1A, helper virus, and HDAd (pC4HSU) are reported in Table S2.

### 2.3. Surgical Protocol

Rabbits were anesthetized with intramuscular ketamine and xylazine induction, followed by inhaled isoflurane. Following a midline neck incision, a 4–4.5 cm segment of the right common carotid was temporarily isolated from the circulation with vascular clips, and an arteriotomy was performed between the clips. The isolated carotid lumen was then rinsed with DMEM (ThermoFisher, Waltham, MA, USA, 11965092), filled with HDAd (2 × 10^11^ vp/mL in DMEM), and incubated for 20 min, transducing a 3–3.5 cm region of the carotid [37]. After the incubation, luminal content was aspirated and flushed, the arteriotomy repaired, and blood flow re-established. The procedure was then repeated on the left common carotid artery. Each rabbit received HDAdAntimiR (*n* = 11 rabbits; *n* = 5 males and *n* = 6 females) or HDAdXMoAntimiR (*n* = 11 rabbits; *n* = 6 males and *n* = 5 females) on one side, whereas the corresponding control vector, HDAdScr or HDAdXMoScr, was infused in the opposite side, with the artery receiving each vector randomized based on a random number-generating algorithm (Excel).

In a previous study, we found that transgene expression in the rabbit carotid reaches a maximum by 3 days after HDAd infusion [16]. Thus, we selected this time frame for the current study. Three days after HDAd treatment, rabbits were placed under general anesthesia. The neck was re-opened with a midline incision and silk ligatures were used to isolate the transduced carotid segments. These segments were excised (the transduced carotid segment contracts to ~2 cm after excision), flushed gently with saline, and cut into 5 smaller segments designated 1–5 from the cranial to the caudal end (Figure S2). Segments 1, 3, and 5 (3 mm each) were embedded in an OCT medium (1 block per artery containing all the 3 segments) and frozen on dry ice. Segments 2 and 4 (5 mm each) were enzymatically digested and the cell suspension was processed for fluorescence-activated cell sorting, as described below. Samples from the left lateral, left medial, and right lobes of rabbit livers were snap-frozen in liquid nitrogen for later RNA extraction.

### 2.4. Fluorescence-Activated Cell Sorting

**Figure 1:**
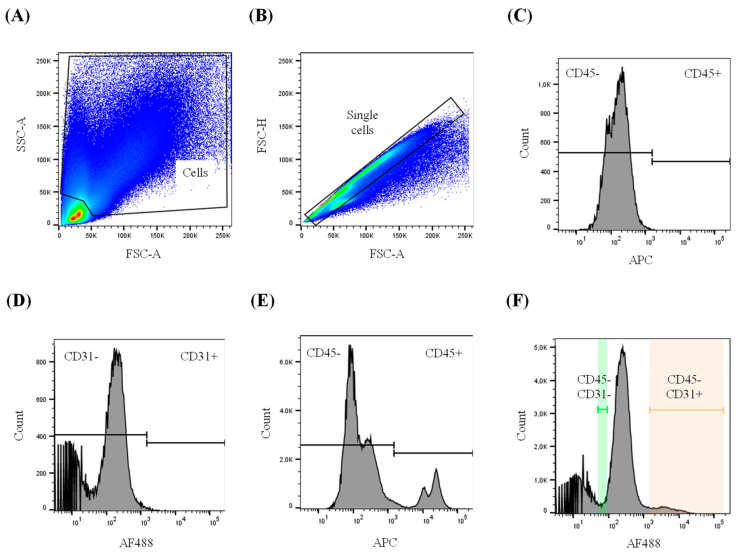
Sorting of carotid endothelial and smooth muscle cells. Three days after HDAd treatment, rabbit carotids were harvested and enzymatically digested, and cell suspensions were stained with antibodies targeting CD45 (immune cell marker) and CD31 (endothelial cell marker) or isotype controls. (**A**,**B**) Scatter plots of (**A**) total cells and (**B**) single cells were used to discard potential cell debris and doublet events. Each dot represents one event; the red color indicates a higher concentration of events, whereas blue represents less event concentration. The graph axes show SSC and FSC (arbitrary units) that represent cellular granularity and size, respectively. (**C**–**F**) Histograms show the fluorescence intensity from APC or AF488 fluorochromes (logarithmic scale, arbitrary units) on the X-axis and event count on the Y-axis. Gates for (**C**) CD45 and (**D**) CD31 were set using isotype control antibodies. (**E**) Histogram of CD45 staining allowed for discrimination of CD45-negative cells—primarily endothelial cells and smooth muscle cells—from CD45-positive cells (i.e., immune cells). (**F**) Histogram of CD31 staining using the previously gated (**E**) CD45-negative cell population. The CD45−CD31+ endothelial cells (shown with red background) and CD45−CD31− smooth muscle cells (green background) were sorted for downstream RNA analysis. To minimize the potential sorting of EC (with lower CD31 expression than the established CD31+ threshold) into the SMC-enriched CD45−CD31− population, the gate for CD45−CD31− cells (green background) was set at the lowest AF488 signal of the peak of CD45−CD31− cells. Gate range of EC population (AF488, arbitrary units): 2 × 10^3^ to 2 × 10^5^. Gate range of SMC population (AF488, arbitrary units): 5 × 10^1^ to 1 × 10^2^. SSC-A: side scatter area; FSC-A: forward scatter area; FSC-H: forward scatter height; APC: allophycocyanin; AF488: Alexa Fluor 488.

Freshly isolated carotid segments 2 and 4 (Figure S2) were cut into pieces of about 1 mm^3^ and digested for 45 min at 37 °C with gentle shaking every 5 min. The digestion buffer contained 2 U/mL liberase (Roche, Basel, Switzerland, 05401127001) and 2 U/mL elastase (Worthington, Lakewood, NJ, USA, LS002279) in Hanks’ Balanced Salt Solution (HBSS; ThermoFisher, 14025092). Cell suspensions were filtered through 70 μm strainers (Corning, Somerville, MA, USA, 352350) and washed twice with HBSS without calcium and magnesium (ThermoFisher, 14175095) with 10% fetal bovine serum (HyClone, Logan, UT, USA, sh30396.03). Cells were blocked with 5% normal mouse serum (Sigma, Saint Louis, MO, USA, NS03L) and then stained with 1 µg/mL Alexa Fluor (AF) 647 mouse anti-CD45 (ThermoFisher, MA5-28392), 10 µg/mL AF488 mouse anti-CD31 (SCBT, Dallas, TX, USA, sc-376764), and 1 µg/mL chicken anti-ABCA1 (Pacific Immunology, custom made) primary antibodies. The CD45 antibody was used to remove the CD45+ immune cells, whereas the CD31 antibody allowed for the separation of endothelial cells (CD45−CD31+) and smooth muscle cells (CD45−CD31−). The antibody targeting CD45 was labeled with AF647 using Zip Rapid Antibody Labeling Kit (ThermoFisher, Z11235) following the manufacturer’s instructions. Cells were also stained with isotype control antibodies: 1 µg/mL AF647 mouse IgG1 (SCBT, sc-24636), 10 µg/mL AF488 mouse IgG1 (SCBT, sc-3890), and 1 µg/mL chicken IgY (Novus Biologicals, Toronto, ON, Canada, AB-101-C). After labeling with primary or isotype antibodies, cells were stained with the secondary antibody 3 µg/mL PE anti-chicken IgY (Southern Biotech, Birmingham, AL, USA, 6100-09). For each artery, the same number of CD45−CD31+ cells and CD45−CD31− cells were sorted on a BD FACSAria III sorter (BD Biosciences). The gating strategy for the selection of the different cell populations is shown in Figure 1. Sorted cells were collected in DNA/RNA shield buffer (Zymo, Irvine, CA, USA, R1100) and cell extracts were used for downstream RNA analysis as explained below. Additionally, ABCA1 protein was quantified as the median fluorescence intensity with FlowJo software (Tree Star Inc., Ashland, OR, USA, version 10.5).

### 2.5. Laser Microdissection (LMD)

**Figure 2:**
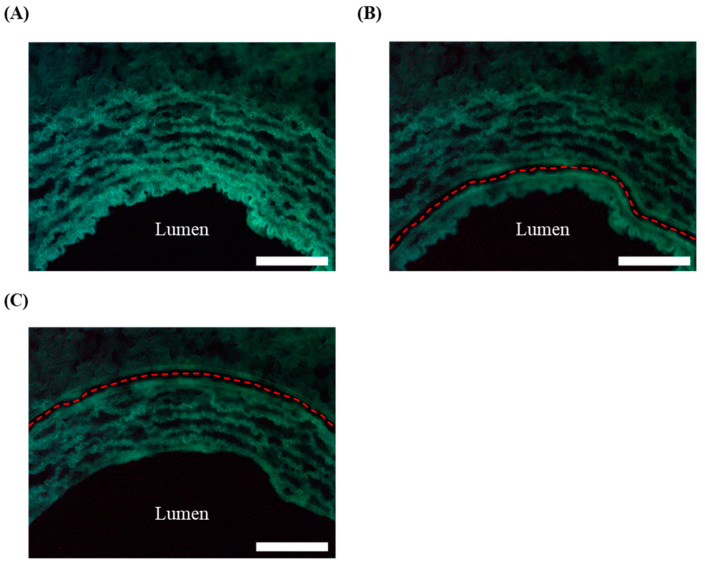
Fluorescence images of a representative rabbit carotid section used for laser microdissection of the intima and media. (**A**) Non-dissected carotid, (**B**) dissection of the intima, and (**C**) dissection of the media. The dotted red line represents the trace followed by the laser. The lumen of the artery is indicated. The scale bar corresponds to 100 µm.

OCT blocks (each containing 3 artery segments per carotid) were sectioned (10 μm thick) onto 4 µm polyethylene naphthalate (PEN) membrane glass slides (Leica, Nussloch, Germany, 11600288). Before the LMD procedure, tissue sections were fixed by subsequent immersion in 75%, 95%, and 100% (*v*/*v*) ethanol solutions for 30 s each. Using the LMD6500 system (Leica), the intima and media from all carotid arteries were microdissected and collected separately in a DNA/RNA shield buffer (Zymo, R1100). First, intima was microdissected by cutting the carotid tissue underneath the endothelial layer, keeping a safe distance (approximately 30 µm) from the luminal endothelium to avoid laser-associated damage to the ECs. Second, the media region of the artery was microdissected (approximately 100 µm) based on the autofluorescence of the elastic lamina (Figure 2). In total, 36 carotid sections were microdissected from each artery and were stored at −80 °C until DNA or RNA extraction, as explained below.

### 2.6. Measurement of Vector DNA

Total DNA was extracted from the microdissected intima and media samples using the Quick-DNA kit (Zymo, D3020). Each DNA extraction required the intima and media from 12 carotid sections. Additionally, 20 mg of rabbit liver (pool of 3 lobules) was pulverized with a dry-ice-cooled mortar and pestle. DNA from the rabbit liver was extracted using the Quick-DNA kit (Zymo, D3024) and quantified by spectrophotometry (NanoDrop, ThermoFisher). A serial dilution of rabbit liver DNA was used to quantify diploid cells, while a serial dilution of the pC4HSU plasmid (Microbix) was utilized for vector genome quantification. Multiplexed real-time quantitative PCR was conducted in duplicate (i.e., two technical replicates) for each intima and media sample to measure the rabbit genomic GAPDH and the non-coding stuffer DNA of the HDAd backbone. The vector copy number per intimal and medial cell was calculated by dividing the number of vector genomes by the number of diploid cells. Primer and probe sequences are provided in Table S2.

### 2.7. Measurement of RNA

Total RNA from the sorted cells and the microdissected samples was extracted with the Quick-RNA kit (Zymo, R1050). For the sorted cells, each RNA extraction was performed with samples containing between 10,000 and 25,000 cells. For the microdissected samples, each RNA extraction required the intima and media from 24 carotid sections. In addition, 20 mg of rabbit liver (pool of 3 lobules) was pulverized with a dry-ice-cooled mortar and pestle. Total RNA from the liver was extracted with the Quick-RNA kit (Zymo, R1054). The entire RNA volume was used for the reverse transcription of both the sorted cells and the microdissected samples. For liver samples, 100 ng of RNA (measured by spectrophotometry with NanoDrop) was used for the reverse transcription. Target mRNA and small RNA (i.e., miRNAs and antagomirs) were measured by real-time quantitative PCR. Custom primers (IDT, Newark, NJ, USA) were used for measuring mRNA targets, while locked nucleic acid primers (QIAGEN, Hilden, Germany) were used for small RNA quantification (primer and probe sequences are found in Tables S2 and S3). Relative gene expression was calculated using the delta-delta Cq method [38]. Standard curves with synthetic oligonucleotides of miR-33a-5p (Bioneer, Daejeon, South Korea), anti-miR-33a-5p (IDT), and XMo-anti-miR-33a-5p (IDT) were used for quantifying absolute target expression and defining the detection limit of the PCR assay (Figure S3). The PCR results were normalized by GAPDH mRNA or snU6 small RNA. For the cell sorting experiment, the number of sorted cells was used for normalizing gene expression. All the PCR targets were measured in two independent wells (i.e., two technical replicates per sample) and the mean value of both wells was calculated. To improve the accuracy of the quantification of small RNA, the standard deviation of both technical replicates was required to be lower than 0.5 Cq. Because of technical impediments, the RNA from sorted cell extracts of 4 out of 22 rabbits (*n* = 2 HDAdAntimiR-treated and *n* = 2 HDAdXMoAntimiR-treated) was not available.

### 2.8. Statistical Analysis

The results are reported as mean ± standard deviation or median (25–75% interquartile range), as indicated. Sample size calculation of the animal study was estimated to detect at least a 1.7-fold difference in transgene expression based on a previous in vivo study with the HDAdABCA1 vector (unpublished data). Data normality was assessed with the Shapiro–Wilk test. When comparing two groups of paired samples, we performed a *t*-test if the normality assumption was met or, in turn, the non-parametric Wilcoxon signed-rank test. When comparing two groups of independent samples, we used a *t*-test for normally distributed data or a Wilcoxon rank-sum test as the non-parametric alternative. For comparing more than two groups of independent samples, we performed the Kruskal–Wallis test followed by Dunn’s test with Bonferroni adjustment for multiple comparisons. Categorical data for independent samples were analyzed with Fisher’s exact test. For paired samples, McNemar’s exact test was employed. Statistical significance was established as *p* < 0.05. All tests were performed with STATA (College Station, TX, USA, version 13.1).

## 3. Results

### 3.1. Anti-miR-33a-5p Is Expressed in the Intima of HDAd-Transduced Carotids

We previously reported the expression of a miR-33a-5p-targeted antagomir (anti-miR-33a-5p) in HDAd-transduced endothelial cells in vitro [33]. To test whether the anti-miR-33a-5p can also be expressed by arterial cells in vivo, we intraluminally infused rabbit carotids with an HDAd expressing anti-miR-33a-5p (HDAdAntimiR) or a scrambled antagomir (HDAdScr). We harvested HDAd-transduced arteries after 3 days and separated intima and media using laser microdissection (Figure 2).

To assess the separation of intima and media, the endothelial (CDH5) and the SMC (ACTG2) markers were analyzed by qPCR. Intima samples from both HDAdAntimiR- and HDAdScr-transduced carotids were significantly enriched in ECs (CDH5/ACTG2 ratio > 379-fold, *p* < 0.001 for both HDAd vectors) compared to the SMC-enriched media samples (Figure S4A,B).

We then evaluated the transduction efficacy of the HDAd-infused arteries. Vector genomes in the intimal cells were significantly higher than in the medial cells in both HDAdAntimiR- (2.6-fold, *p* = 0.004) and HDAdScr-treated arteries (58.6-fold, *p* = 0.003), indicating that anti-miR-33a-5p and scrambled antagomir were primarily expressed by the carotid endothelium (Figure S5A,B). This is in accordance with our previous report showing that HDAd-encoded transgenes are almost exclusively expressed by luminal ECs after local carotid infusions [39]. Surprisingly, the HDAdAntimiR-transduced arteries presented 34-fold fewer (*p* < 0.001) vector genomes in the intima than HDAdScr-transduced arteries (Figure S5C,D), which may indicate an issue with the transduction efficacy of the HDAdAntimiR vector.

**Figure 3:**
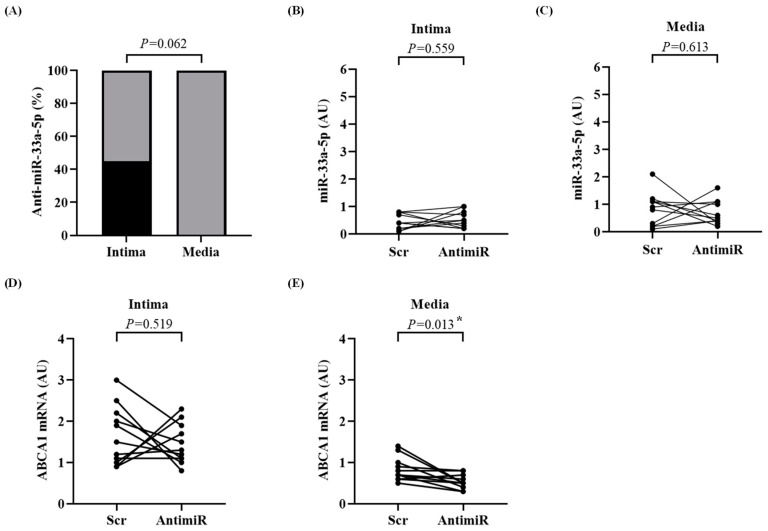
Expression of anti-miR-33a-5p, miR-33a-5p, and ABCA1 mRNA in the intima and media. Arteries were harvested 3 days after treatment with HDAdScr (Scr) or HDAdAntimiR (AntimiR). Intima and media were microdissected and the antagomir, miR-33a-5p, and ABCA1 mRNA were quantified by real-time quantitative PCR. (**A**) Percentage of carotid intima and media samples in which anti-miR-33a-5p was detected. The percentage of detected samples is shown in black and non-detected samples in gray. Each intima and media sample is from one artery. *n* = 11 intima and media. *p* value from McNemar’s exact test. (**B**,**C**) Expression of miR-33a-5p in the (**B**) intima and (**C**) media of HDAdScr-infused arteries compared to HDAdAntimiR treatment. (**D**,**E**) Expression of ABCA1 mRNA in the (**D**) intima and (**E**) media of HDAdScr- or HDAdAntimiR-treated arteries. Each data point is from the intima or media of one carotid; data points from the same rabbit are connected by a line. *n* = 11 carotid intima and media per HDAd vector. *p* values are from paired *t*-tests. * *p* < 0.05 vs. Scr. HDAd: helper-dependent adenovirus; AUs: arbitrary units.

Anti-miR-33a-5p was expressed within the intima of 45% of the carotids transduced with HDAdAntimiR (5 out of 11, *p* = 0.062), whereas no expression was detected within the media (Figure 3A). In turn, anti-miR-33a-5p was not detected in either the intima or media of HDAdScr-transduced arteries. We subsequently assessed whether anti-miR-33a-5p expression in the carotid arteries could affect the levels of miR-33a-5p, as well as ABCA1 mRNA, since ABCA1 is post-transcriptionally regulated by miR-33a-5p. Overall, the transcript levels of miR-33a-5p and ABCA1 were not altered in either the intima or media of the transduced carotids, suggesting that the levels of anti-miR-33a-5p were insufficient to exert a detectable biological effect (Figure 3B–E). Unexpectedly, there was a small decrease in ABCA1 mRNA (1.6-fold, *p* = 0.013) in the media of HDAdAntimiR-treated arteries compared to the paired scramble control (Figure 3E).

### 3.2. X-Motif Anti-miR-33a-5p Is Detectable in the Media of a Small Percentage of Carotids

Our results indicate that the expression of anti-miR-33a-5p is limited to the intima of HDAdAntimiR-transduced carotid arteries. We previously reported that the addition of an X-motif to the anti-miR-33a-5p (XMo-anti-miR-33a-5p) promotes its loading into endothelial cell-derived exosomes and its transfer to vascular SMCs in vitro [33]. Given that the SMCs are the predominant cell type in the artery media, we evaluated whether the X-motif also enhances anti-miR-33a-5p levels in the medial region. We infused rabbit carotids intraluminally with an HDAd expressing X-motif-anti-miR-33a-5p (HDAdXMoAntimiR) or the X-motif-scrambled antagomir (HDAdXMoScr). Arteries were harvested 3 days after transduction and intima and media were separated by laser microdissection.

Intima extracts were significantly enriched in ECs (CDH5/ACTG2 ratio > 763-fold and *p* = 0.003 for both HDAd vectors) compared to media samples which were enriched in SMCs (Figure S4C,D). The HDAd vector genomes were significantly higher in the intima than in the media (>23-fold and *p* = 0.003 for both HDAd vectors, Figure S6A,B). Moreover, the transduction of carotids with HDAdXMoAntimiR was similar to HDAdXMoScr transduction in both the intima (*p* = 0.224) and media (*p* = 0.094) (Figure S6C,D).

**Figure 4:**
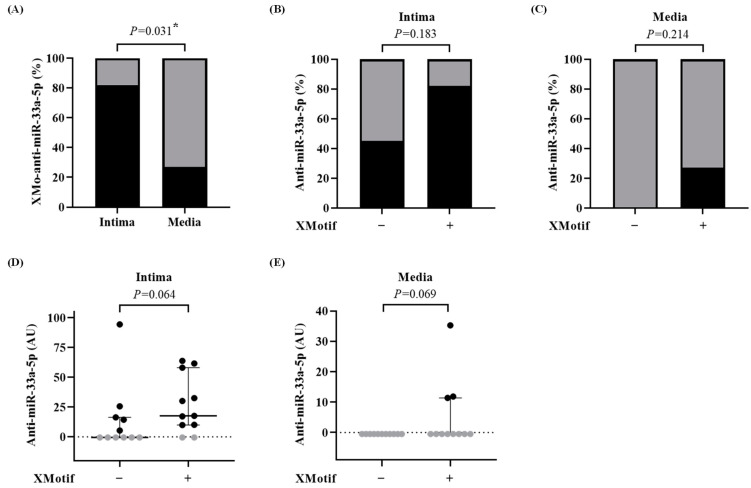
Expression of X-motif anti-miR-33a-5p in the intima and media. Arteries were harvested 3 days after treatment with HDAdAntimiR or HDAdXMoAntimiR. Intima and media were microdissected and the antagomirs, without or with the X-motif, were quantified by real-time quantitative PCR. (**A**) Percentage of carotid intima and media samples in which XMo-anti-miR-33a-5p was detected. The percentage of detected samples is shown in black and non-detected samples in gray. Each intima and media sample is from one artery. *n* = 11 intima and media. *p* value from McNemar’s exact test. * *p* < 0.05 vs. intima. (**B**,**C**) Percentage of carotid (**B**) intima and (**C**) media in which the antagomir was detected, either without (−) or with (+) the X-motif. *n* = 11 intima and media per HDAd vector. *p* values from Fisher’s exact test. (**D**,**E**) Expression of the antagomir, without and with the X-motif, in the (**D**) intima and (**E**) media of HDAd-treated vessels. Target expression was normalized to the snU6 reference gene. Data points represent individual arteries. Bars and whiskers are group medians and interquartile ranges, respectively. The dotted line is the PCR assay’s limit of detection (see Figure S3); data points below the detection limit are shown in gray. *n* = 11 intima and media per HDAd vector. *p* values are from the Wilcoxon rank-sum test. HDAd: helper-dependent adenovirus; AUs: arbitrary units.

The XMo-anti-miR-33a-5p was expressed in 82% of the intimal extracts (9 out of 11 carotids) but only 27% of medial samples (3 out of 11 carotids, *p* = 0.031) had detectable expression of the X-motif antagomir (Figure 4A). The X-motif antagomir was not detected in either the intima or media of carotids treated with HDAdXMoScr.

**Figure 5:**
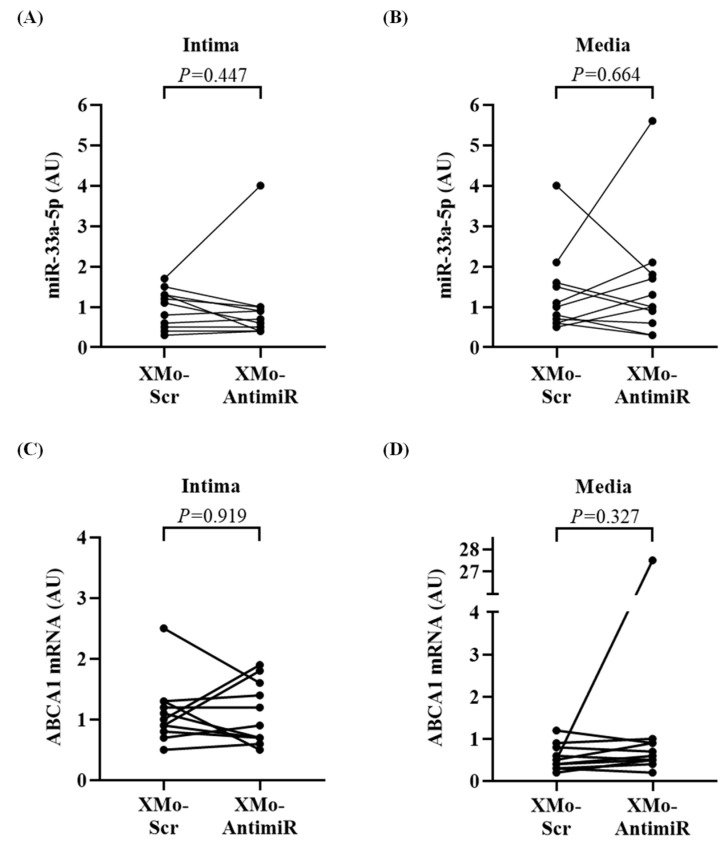
Effect of X-motif antagomir on miR-33a-5p and ABCA1 mRNA expression. Intima and media were microdissected 3 days after treating rabbit carotids with HDAdXMoScr (XMo-Scr) or HDAdXMoAntimiR (XMo-AntimiR). The expression of miR-33a-5p and ABCA1 mRNA was measured by qPCR. (**A**,**B**) Expression of miR-33a-5p in the (**A**) intima and (**B**) media of HDAdXMoScr-treated arteries compared to HDAdXMoAntimiR. (**C**,**D**) Expression of ABCA1 mRNA in the (**C**) intima and (**D**) media of HDAdXMoScr-treated or HDAdXMoAntimiR-infused arteries. Each data point is from the intima or media of one carotid; data points from the same rabbit are connected by a line. *n* = 11 carotid intima and media per HDAd vector. *p* values are from (**A,D**) Wilcoxon signed-rank tests and (**B**,**C**) paired *t*-tests. HDAd: helper-dependent adenovirus; qPCR: real-time quantitative PCR; AUs: arbitrary units.

We then compared the expression of both antagomirs—with and without the X-motif—in the HDAdXMoAntimiR- and HDAdAntimiR-transduced carotids. The X-motif antagomir was expressed in the intima (*p* = 0.183) and media (*p* = 0.214) of more carotids than the non-X-motif antagomir (Figure 4B–C). Moreover, the expression of the X-motif antagomir was 2-fold higher in the intima (*p* = 0.064) compared to non-X-motif antagomir (Figure 4D), which may be explained by the higher vector genomes in the HDAdXMoAntimiR-transduced carotids (Figure S6E,F). The expression of X-motif antagomirs was also higher in the media (*p* = 0.069) than in the non-X-motif antagomir (Figure 4E). Despite the higher expression of X-motif antagomir in the transduced arteries, the levels of miR-33a-5p and ABCA1 mRNA were not affected in either the intima or media (Figure 5A–D).

### 3.3. Antagomir Expression Is Suboptimal in Both ECs and SMCs

Overall, our results show that anti-miR-33a-5p, with or without the X-motif, is predominantly contained within the intima of HDAd-transduced arteries. The arterial intima is primarily composed of ECs and, to a lesser extent, SMCs, which are the major constituents of the medial region. To further characterize the cellular distribution of the antagomir, we analyzed its expression in endothelial cell-enriched (CD45−CD31+) and smooth muscle cell-enriched (CD45−CD31−) sorted cell fractions from arteries transduced with either HDAdAntimiR or HDAdXMoAntimiR. We harvested HDAd-transduced arteries 3 days after infusions and separated the EC-enriched and SMC-enriched populations using a cell sorter.

First, we evaluated the expression of cell-type markers (i.e., CDH5 and ACTG2) in the sorted cells. The CD45−CD31+ population was significantly enriched in ECs (CDH5/ACTG2 ratio > 28-fold and *p* ≤ 0.013 for all the HDAd vectors) in comparison to the SMC-enriched CD45−CD31− population (Figure S7A–D).

**Figure 6:**
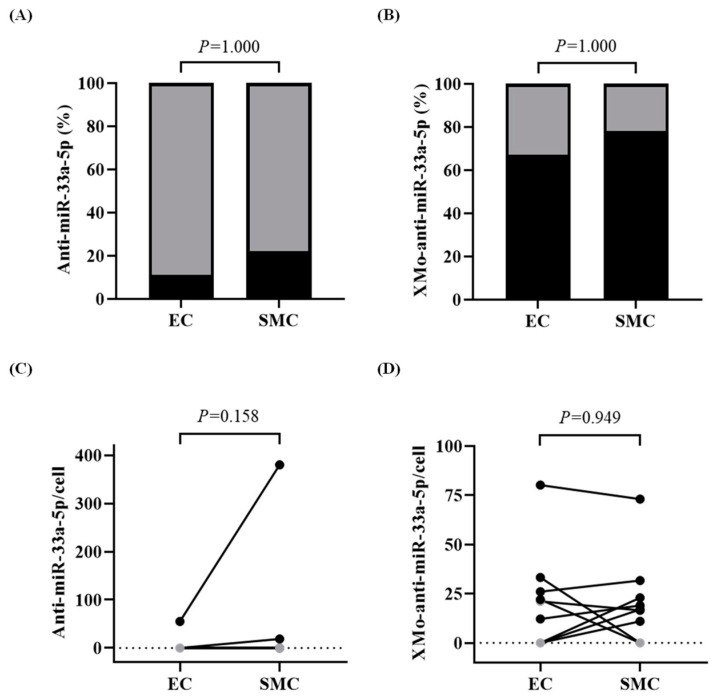
Distribution of antagomirs in the endothelial cells (ECs) and smooth muscle cells (SMCs). Three days after treatment with HDAdAntimiR or HDAdXMoAntimiR, rabbit arteries were removed and enzymatically digested. Cell suspensions were stained with fluorescently labeled antibodies and EC-enriched (CD45−CD31+) and SMC-enriched (CD45−CD31−) populations were sorted for subsequent RNA analysis. (**A**,**B**) Percentage of carotid EC and SMC samples in which (**A**) anti-miR-33a-5p and (**B**) XMo-anti-miR-33a-5p were detected. The percentage of detected samples is shown in black and non-detected samples in gray. Each EC and SMC sample is from one artery. *n* = 9 samples of ECs and SMCs per HDAd vector. *p* values from McNemar’s exact test. (**C**,**D**) Expression of (**C**) anti-miR-33a-5p and (**D**) XMo-anti-miR-33a-5p (transcripts per cell) in the sorted ECs and SMCs. Data points are from individual arteries; points from the same artery are connected by lines. The dotted line is the PCR assay’s limit of detection (see Figure S3); gray data points are below the detection limit. *n* = 9 samples of ECs and SMCs per HDAd vector. *p* values are from (**C**) Wilcoxon signed-rank test and (**D**) paired *t*-test. HDAd: helper-dependent adenovirus.

The anti-miR-33a-5p (without the X-motif) was lowly expressed and only detected in the ECs of 11% of the carotids (1 out of 9) and in the SMCs of 22% of the carotids (2 out of 9, Figure 6A,C). In contrast, the XMo-anti-miR-33a-5p was detected in the ECs of 67% of the carotids (6 out of 9) and in the SMCs of 78% of the carotids (7 out of 9, Figure 6B). Moreover, the levels of XMo-anti-miR-33a-5p were similar (*p* = 0.949) in both the ECs and SMCs (Figure 6D). None of the HDAd-scrambled–transduced arteries, with or without the X-motif, had detectable antagomir expression.

**Figure 7:**
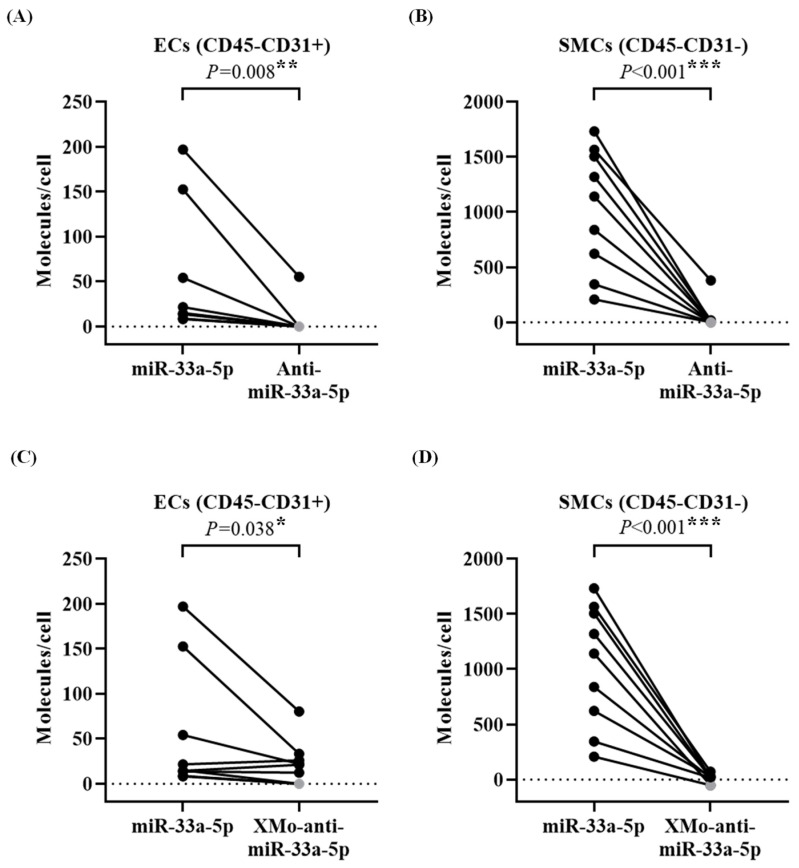
Stoichiometry of the antagomir and miR-33a-5p in the endothelial cells (ECs) and smooth muscle cells (SMCs). Rabbit arteries were harvested 3 days after treatment with HDAdAntimiR or HDAdXMoAntimiR. Vessels were enzymatically digested, and cell suspensions were stained with antibodies. The EC-enriched (CD45−CD31+) and SMC-enriched (CD45−CD31−) populations were sorted for RNA analysis. (**A**,**B**) Quantification of the miR-33a-5p and anti-miR-33a-5p (molecules per cell) in the sorted (**A**) EC and (**B**) SMC extracts. (**C**,**D**) Expression of the miR-33a-5p and XMo-anti-miR-33a-5p (molecules per cell) in the sorted (**C**) EC and (**D**) SMC samples. Data points represent individual arteries; points from the same artery are connected by lines. The dotted line is the PCR assay’s limit of detection (see Figure S3); data points below the detection limit are shown in gray. *n* = 9 samples of ECs and SMCs per HDAd vector. *p* values are from (**A**,**C**) Wilcoxon signed-rank tests and (**B,D**) paired *t*-tests. * *p* < 0.05, ** *p* < 0.01, and *** *p* < 0.001 vs. miR-33a-5p. HDAd: helper-dependent adenovirus.

Antagomirs exert their inhibitory function via binding to their targets with a one-to-one stoichiometry [40,41]. To determine whether the cellular antagomir level is sufficient to inhibit miR-33a-5p, we compared their expression in the sorted ECs and SMCs. The non-X-motif antagomir showed significantly fewer transcripts (*p* ≤ 0.008) compared to miR-33a-5p in both ECs (at least 2.1-fold) and SMCs (at least 20.3-fold) of the HDAdAntimiR-infused vessels (Figure 7A,B). Similarly, XMo-anti-miR-33a-5p was 2.5-fold lower (*p* = 0.038) than miR-33a-5p in the ECs and 48.5-fold lower (*p* < 0.001) within the SMCs of HDAdXMoAntimiR-transduced arteries (Figure 7C,D).

Our results (Figure 7A–D) suggest that antagomir expression, both without and with the X-motif, is below the optimal stoichiometry to inhibit miR-33a-5p. Accordingly, the expression of miR-33a-5p in ECs and SMCs was not appreciably changed by either of the antagomirs compared to the paired scramble controls (Figure S8A–D). Similarly, the expression of ABCA1 mRNA and its protein level were unchanged by the antagomirs in both EC and SMC samples (Figures S9A–D and S10A–D).

### 3.4. Antagomirs Do Not Accumulate in the Rabbit Liver

Endothelial cells release exosomes either through their apical or basolateral sides [42]. Accordingly, the HDAd-transduced endothelial cells may release the antagomir-loaded exosomes either toward the artery lumen (apically) or the subendothelium (basolaterally). The circulating blood exosomes predominantly accumulate in the liver, where they are internalized by Kupffer cells and other hepatic cell types [43]. To evaluate whether anti-miR-33a-5p, either with or without the X-motif, accumulates within rabbit livers, we measured its expression 3 days after HDAd infusions.

**Figure 8:**
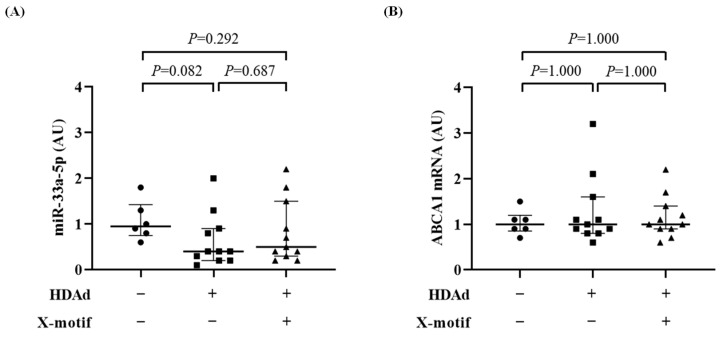
Lack of off-target downstream effects of antagomirs in the liver. Rabbit livers were collected from non-HDAd-treated (−) and HDAd-treated (+) rabbits. The HDAd vectors encoded the anti-miR-33a-5p or a scrambled antagomir, either without (−) or with (+) the X-motif. Livers were harvested 3 days after HDAd treatment and RNA was extracted from pooled left, right, and middle lobules. (**A**,**B**) Liver expression of (**A**) miR-33a-5p and (**B**) ABCA1 mRNA; target expression was normalized with the snU6 and GAPDH mRNA, respectively. Data points represent individual livers. Bars and whiskers are group medians and interquartile ranges, respectively. *n* = 6 livers from non-HDAd-treated rabbits. *n* = 22 livers from HDAd-treated rabbits, either without (*n* = 11) or with (*n* = 11) the X-motif. *p* values are from Dunn’s test adjusted by Bonferroni. HDAd: helper-dependent adenovirus; AUs: arbitrary units.

We did not detect any antagomir, with or without the X-motif, in the livers of rabbits infused with HDAd. Moreover, no significant changes were observed in the expression of miR-33a-5p or ABCA1 mRNA in the livers of experimental rabbits when compared to control rabbits not infused with HDAd (Figure 8A,B).

## 4. Discussion

We transduced rabbit carotid endothelium with an anti-miR-33a-5p-expressing HDAd and tested whether the X-motif could enhance antagomir transfer to the medial cells. Our major findings were as follows: (1) the anti-miR-33a-5p was expressed in the artery intima 3 days after HDAd-infusion; (2) the X-motif antagomir was detected in 82% of intima extracts and in the media of 27% of carotids; (3) the expression of anti-miR-33a-5p, with and without the X-motif, was suboptimal in both ECs and SMCs, and it did not alter miR-33a-5p and ABCA1 levels; and (4) the antagomirs were not detected in the rabbit liver 3 days after treatment.

Cholesterol accumulation in the artery wall is a major driver of atherosclerosis [44]. MiR-33a-5p is a pro-atherogenic miRNA that represses genes involved in cholesterol metabolism (e.g., ATG5) and efflux (e.g., ABCA1), leading to increased intracellular cholesterol [23,25]. Both the miR-33a-5p sequence and its regulation of ABCA1 are highly conserved, which enhances the translational potential [45,46]. The inhibition of miR-33a-5p promotes cholesterol efflux, raises plasma HDL levels, and enhances RCT from arteries [47]. Accordingly, miR-33a-5p inhibition is considered a promising therapy for treating atherosclerosis [27,47,48]. The strategy conducted here allowed the in vivo expression of anti-miR-33a-5p antagomirs directly by the transduced arterial endothelium. Anti-miR-33a-5p expression, without the X-motif, was confined to the vessel intima, with no detectable antagomir expression in the media. Moreover, the expression of miR-33a-5p and ABCA1 mRNA in the artery was not altered, suggesting that the antagomir level was suboptimal. Our approach contrasts with prior investigations utilizing a synthetic anti-miR-33a-5p that is administered systemically [47,48,49]. In these studies, the synthetic antagomir promoted regression of atheroma but failed to alter its progression in low-density lipoprotein receptor-deficient (*Ldlr−/−*) mice. In addition to its limited efficacy, the systemically administered antagomir accumulated in the murine liver and other peripheral organs, leading to long-term adverse effects [47,48,49]. Compared with these precedents, our approach of expressing antagomirs by the transduced arterial ECs has several theoretical advantages. First, it provides the antagomir directly to the artery in which it is needed. Second, HDAd-mediated in vivo transduction allows sustained transgene expression in the ECs for at least 48 weeks, avoiding the need for frequent antagomir infusions [16]. Third, the localized antagomir expression minimizes the risk of adverse effects associated with miR-33a-5p inhibition via off-target tissues [28].

The expression of anti-miR-33a-5p in the ECs could serve as a promising strategy for treating atherosclerosis, as lipid accumulation within ECs plays a key role in atherogenesis [50]. However, cholesterol buildup in the intimal macrophages and SMCs also significantly contributes to the progression of atherosclerosis [51]. Accordingly, transferring the anti-miR-33a-5p to the subendothelial cells—macrophages and SMCs—could theoretically potentiate the anti-atherosclerotic effects of the antagomir. This hypothesis is supported by previous reports in which miR-33a-5p deficiency in macrophages led to increased ABCA1 protein and cholesterol efflux capacity [23]. Additionally, Price et al. showed that a murine bone marrow transplant from miR-33a knockout mice into *Ldlr−/−* mice impedes the development of atherosclerotic plaques, suggesting that macrophage-specific inhibition of miR-33a-5p could be a promising therapy [27]. The effect of miR-33a-5p deficiency in SMCs is less well understood, although in vitro data suggest that it leads to increased ABCA1 protein and a variable cell line-dependent effect on cholesterol efflux [33,52].

Strategies for the targeted delivery of antagomirs to the vasculature include viral vectors, nanoparticles, and exosomes [53]. The artery endothelium is easily accessible to these delivery systems, whereas gene transfer to the abluminal cells is less efficient [39,54]. To circumvent this limitation, our strategy exploits exosome-mediated cell-to-cell communication processes. Exosomes are capable of crossing complex biological barriers (e.g., the blood–brain barrier), including the transfer of small RNA between ECs and SMCs in vivo [32,55,56]. Accordingly, antagomir-loaded exosomes that are released from HDAd-transduced luminal ECs could theoretically penetrate into the intima and transfer the antagomir to vascular SMCs and macrophages.

The X-motif promotes the loading of miRNA and other small RNA (e.g., antagomirs) into exosomes [57,58,59]. In a previous study, we added the X-motif to anti-miR-33a-5p, which enhanced antagomir encapsulation into exosomes released from transduced ECs [33]. This system promoted antagomir transfer via EC-derived exosomes into vascular SMCs and macrophages in vitro. Moreover, this exosome-mediated miR-33a-5p inhibition led to increased ABCA1 protein and ApoAI-mediated cholesterol efflux in both tested cell types [33]. In our present study, contrary to our expectations, the inclusion of the X-motif sequence did not significantly enhance antagomir delivery to rabbit carotid artery medial cells, with only 27% of the arteries having detectable antagomir expression within the media. In an apparent contradiction, the level of X-motif antagomir was similar in both EC-enriched and SMC-enriched sorted cell populations. This result may be explained by the less efficient cell-type enrichment of the sorted cell samples (CDH5/ACTG2 ratio of EC vs. SMC: 45-fold, Figure S7D) compared to the laser microdissected samples (CDH5/ACTG2 ratio of intima vs. media: 804-fold, Figure S4D). Nevertheless, both the EC-enriched and SMC-enriched populations presented suboptimal antagomir levels, below one-to-one stoichiometry relative to the miR-33a-5p target, indicating the need to develop a more potent transgene expression strategy. As a likely result of the overall low expression of the antagomirs, the levels of miR-33a-5p and ABCA1 mRNA/protein were not altered in either the EC or SMC populations.

One limitation of this study is that the arteries infused with HDAdAntimir presented significantly fewer vector genomes in the intima than the HDAdScr- and HDAdXMoAntimiR-infused vessels (Figures S5C and S6E). This might have occurred because of a technical issue during vector production that affected the transduction capacity and, unfortunately, we were not able to detect it before the in vivo experiments. The reduced transduction of HDAdAntimir-infused vessels likely led to the overall lower non-X-motif antagomir expression in both laser microdissected samples and sorted cells. The low expression of non-X-motif antagomir could potentially lead to overestimating the effect of the X-motif on antagomir transfer to the medial cells. However, this concern may possibly be dismissed, as the X-motif showed no significant impact on antagomir transfer to the media.

Our in vivo gene therapy approach requires further optimization to enhance antagomir expression and transfer into the artery wall. First, the antagomir expression may be increased by substituting the H1 promoter for the U6 promoter, which may permit more potent transgene expressions within transduced ECs [60]. Second, vectors or alternative delivery methods that better transduce/deliver transgenes to ECs could improve antagomir expression within the vessel wall. In this regard, nanoparticles have the advantages of high biocompatibility, loading capacity, and ease of conjugation with EC-targeting antibodies or ligands [61]. As recently reported, lipid-based nanoparticles loaded with an X-motif-anti-miR-33a-5p-expressing plasmid, which drives this expression with a U6 promoter, efficiently delivered this plasmid DNA to pro-inflammatory ECs in vitro via targeting VCAM-1 [62]. Moreover, this led the pro-inflammatory ECs to robustly package anti-miR-33a-5p within exosomes, which enhanced ABCA1 protein and ApoAI-mediated cholesterol efflux in macrophages exposed to the EC-derived exosomes. Third, the loading of the antagomir into exosomes may be enhanced using novel engineered RNA export systems [63]. For instance, Horns et al. incorporated an aptamer sequence into the RNA molecules that were then encased into protein nanocages and released via small extracellular vesicles [63]. Notably, this approach also allows for the encapsulation of large RNA species, potentially permitting the exosome-mediated transfer of therapeutic mRNAs such as ABCA1 and ApoAI.

Short-term systemic inhibition of miR-33a-5p has atheroprotective effects [47,48], while long-term inhibition has been shown to negatively alter hepatic metabolism, leading to hypertriglyceridemia and hepatic steatosis in mice [28]. Additionally, overexpression of ABCA1 in mouse liver also increases hepatic and plasma cholesterol, which can promote atherosclerosis [64]. ECs release exosomes bidirectionally and direct distinct cargo to apical (circulation) and basolateral (vessel wall) compartments [42]. Our strategy minimizes the risk of adverse effects because the antagomir is expressed locally in the artery wall. Moreover, the X-motif antagomir is primarily released basolaterally from transduced (pro-inflammatory) ECs in vitro [62]. Accordingly, we expected that a low number of antagomir-loaded exosomes would be released apically from the transduced ECs into circulation. However, circulating exosomes tend to accumulate in the liver [43], and thus, antagomir-loaded exosomes may also enter liver cells such as Kupffer cells and hepatocytes. In this regard, we found no detectable antagomir expression, with or without the X-motif, in the rabbit livers 3 days after HDAd infusions. MiR-33a-5p and ABCA1 mRNA were also not altered. However, given the short-term design of this study, we should be cautious and not discard the possibility of side effects regarding a longer-term duration or with higher antagomir expression from the vessel wall.

Developing a successful vascular wall-targeted strategy that allows the delivery of therapeutic antagomirs into the subendothelial SMCs and macrophages could be directly applicable to other diseases beyond atherosclerosis. We speculate that this strategy, if successful, could be translated to treat vascular wall pathologies such as aneurysms and medial calcification in which both miRNA and exosomes play relevant roles [65,66]. Accordingly, patients with chronic kidney disease, diabetes mellitus, or hypertension, common risk factors of vascular disease, could potentially benefit from our exosome-based antagomir delivery system.

## 5. Conclusions

In summary, our gene therapy strategy allowed for the in vivo vascular wall-targeted expression of anti-miR-33a-5p antagomir. However, anti-miR-33a-5p expression was limited to the carotid artery intima, and the X-motif was not sufficiently effective to enhance antagomir transfer into the artery media. Additionally, the antagomir expression was suboptimal, failing to effectively alter miR-33a-5p and ABCA1 levels. Based on all these findings, our approach requires further refinement to improve both the antagomir expression and the exosome-mediated transfer to subendothelial cells. If our anti-miR-33a-5p therapy achieves reasonable efficacy in vivo, this may be combined with vectors overexpressing ApoAI as well as ABCA1 [14,15,16,20], which could enhance atheroprotection via increasing HDL formation locally and potentiating RCT from atherosclerotic arteries.

## Data Availability

The raw data supporting the conclusions of this article will be made available by the authors upon request.

## Supplementary Materials

The following supporting information can be downloaded at https://www.mdpi.com/article/10.3390/biology13120965/s1: Figure S1: Schematic representations of the HDAd expression cassettes; Figure S2: Schematic representation of an HDAd-treated common carotid; Figure S3: Standard curves for the quantification of gene expression; Figure S4: Cell-type markers in the carotid intima and media; Figure S5: HDAd transduction in the intima and media; Figure S6: HDAd vector genomes in the intima and media; Figure S7: Cell-type markers in the sorted carotid endothelial cells (ECs) and smooth muscle cells (SMCs); Figure S8: MiR-33a-5p in the sorted endothelial cells (ECs) and smooth muscle cells (SMCs); Figure S9: Expression of ABCA1 mRNA in the sorted endothelial cells (ECs) and smooth muscle cells (SMCs); Figure S10: ABCA1 protein in the sorted endothelial cells (ECs) and smooth muscle cells (SMCs); Table S1: Expression cassettes of the HDAd vectors; Table S2: Sequences of the primers and probes; Table S3: Target sequences for the quantitative PCR of small RNA.
