# Supplementary material for: Exosome-Mediated Transfer of X-Motif-Tagged Anti-MiR-33a-5p Antagomirs to the Medial Cells of Transduced Rabbit Carotid Arteries
Source: Biology (Basel). 2024 Nov 24;13(12):965. doi: 10.3390/biology13120965 (PMC11673983; doi:10.3390/biology13120965)
Supplement: Supplementary file 1 [file biology-13-00965-s001.zip › biology-3301047-supplementary.pdf]

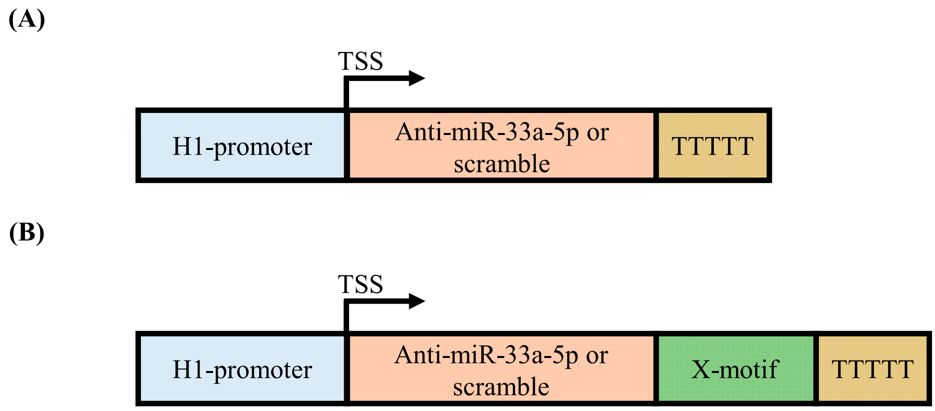

Figure S1. Schematic representations of the HDAd expression cassettes. (**A**) HDAdAntimir and HDAdScr and (**B**) HDAdXMoAntimir and HDAdXMoScr. Transcription is terminated with the 5 consecutive thymidine (T) string. TSS: transcription start site; HDAd: helper-dependent adenovirus.

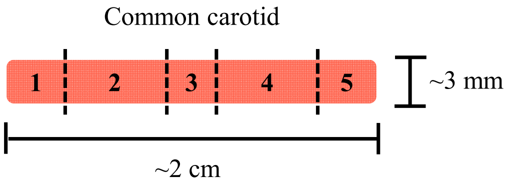

Figure S2. Schematic representation of an HDAd-treated common carotid. 3 days after the infusion of HDAd vectors, the transduced carotids were harvested and cut in 5 smaller segments: number 1, 3 and 5 were embedded in OCT medium and frozen on dry ice, whereas segments 2 and 4 were enzymatically digested and processed for fluorescence activated cell sorting (FACS). HDAd: helper-dependent adenovirus.

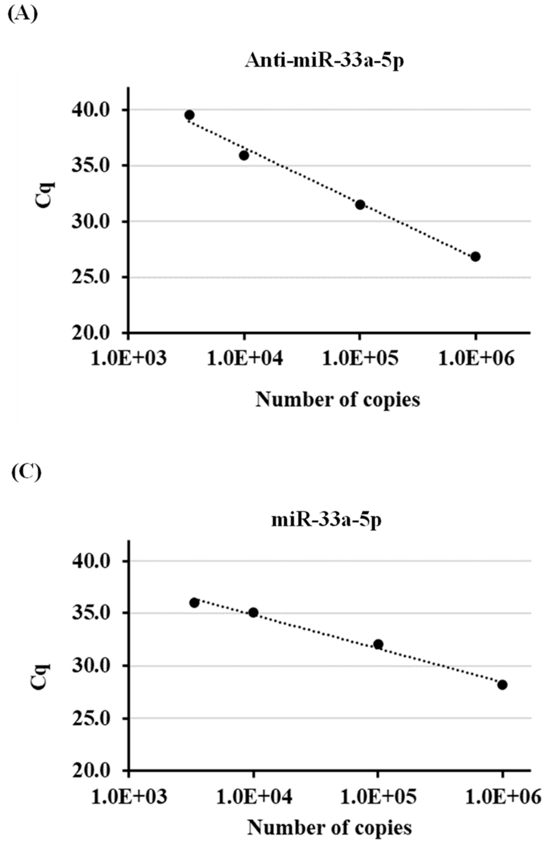

Figure S3. Standard curves for the quantification of gene expression. **(A-C),** standard curves of (**A**) anti-miR-33a-5p, (**B**) XMo-anti-miR-33a-5p and (**C**) miR-33a-5p were analyzed by real-time quantitative PCR. The standard curves were used for estimating the absolute copy number of the target genes. Each data point is the mean value of two PCR wells (technical replicate). The limit of detection for each PCR assay was set at the Cq value corresponding with the lowest detectable copy number; higher Cq values were considered as non-detectable. Standard samples with less than $3.3x10^3$ copies had no detectable PCR signal.

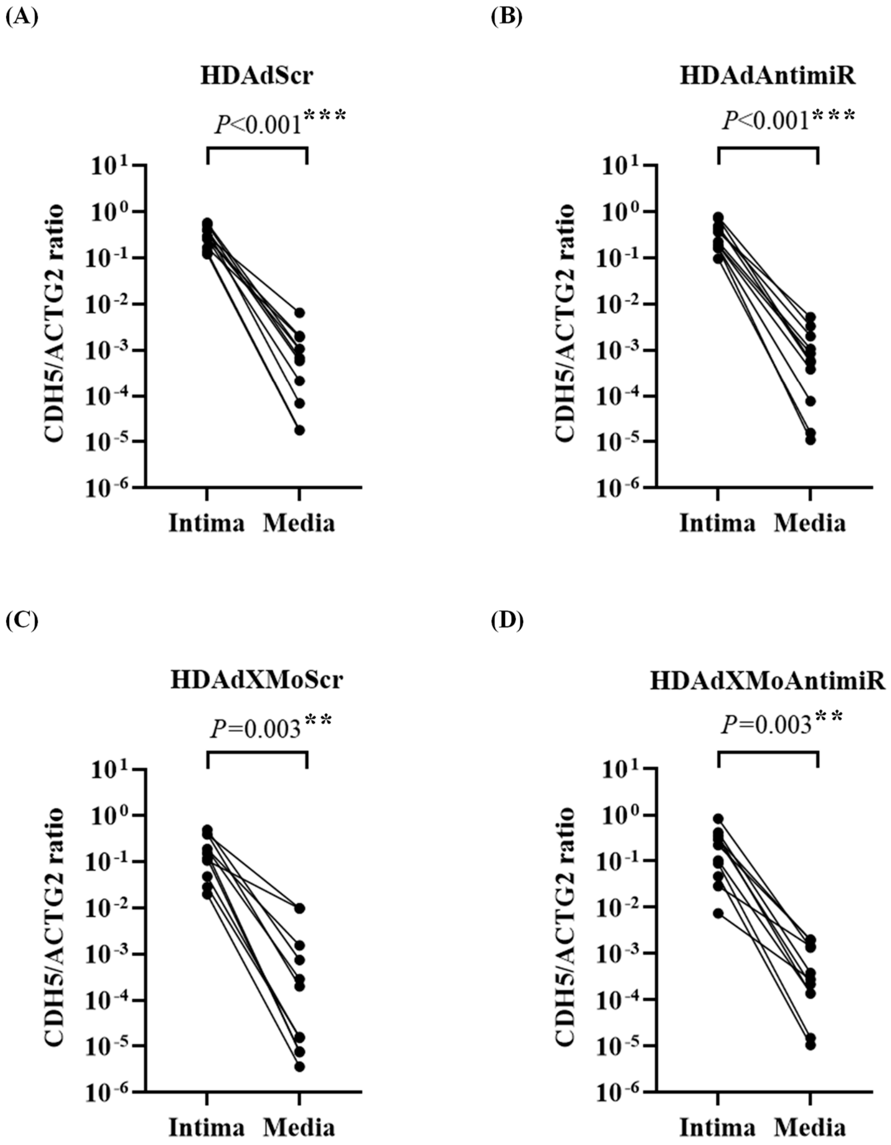

Figure S4. Cell-type markers in the carotid intima and media. Rabbit arteries were treated with (**A**) HDAdScr, (**B**) HDAdAntimiR, (**C**) HDAdXMoScr or (**D**) HDAdXMoAntimiR and were harvested 3 days after treatment. Intima and media of each artery were separated using laser microdissection. The cell-type markers CDH5 (enriched in endothelial cells) and ACTG2 (enriched in smooth muscle cells) were measured in the intima and media extracts by real-time quantitative PCR. Cell-type enrichment was estimated as the ratio between CDH5 and ACTG2 expression; higher ratios indicate enrichment in endothelial cells whereas lower ratios correspond to enrichment in smooth muscle cells. Each data point corresponds to the intima or media from one carotid; data points from the same carotid are connected by a line. N=11 carotid intima and media per HDAd vector. *P* values are from (**A-C**) paired t-test and (**D**) Wilcoxon signed-rank test. ***P*<0.01 and ****P*<0.001 vs Intima. HDAd: helper-dependent adenovirus.

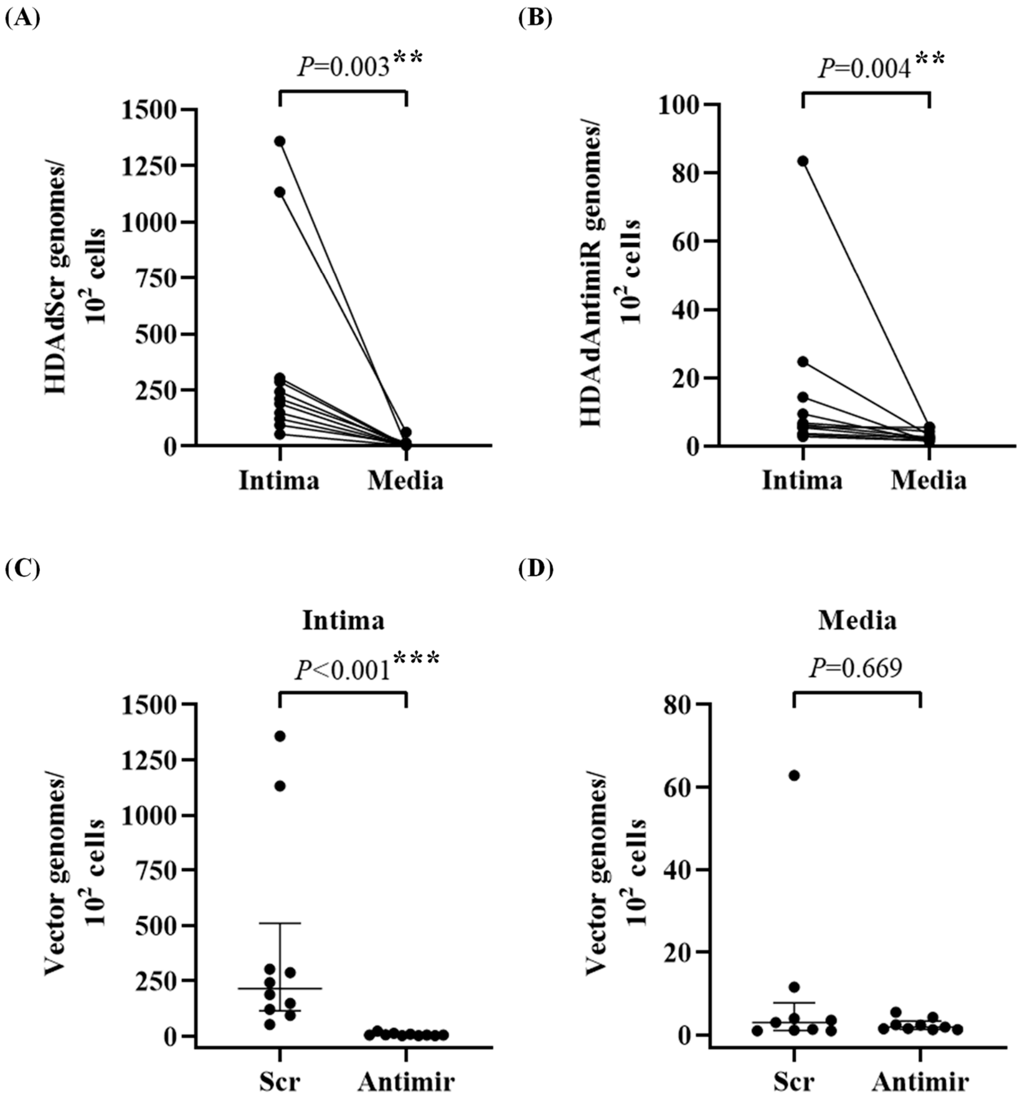

Figure S5. HDAd transduction in the intima and media. Intima and media from rabbit carotids were microdissected 3 days after treatment with HDAdScr (Scr) or HDAdAntimiR (AntimiR). Vector genomes were measured by qPCR in the intima and media extracts and were normalized to the number of diploid cells (expressed as vector genomes per 100 diploid cells). (**A-B),** vector genomes in the intima and media of (**A**) HDAdScr-treated and (**B**) HDAdAntimiR-infused arteries. (**C-D),** HDAd genomes in the (**C**) intima and (**D**) media of arteries treated with either HDAdScr or HDAdAntimiR. Each data point is from the intima or media of one carotid; data points from the same carotid are connected by a line. Bars and whiskers are group medians and interquartile ranges, respectively. N=11 carotid intima and media per HDAd vector. *P* values are from (**A-B**) Wilcoxon signed-rank test and (**C-D**) Wilcoxon rank-sum test. ***P*<0.01 and ****P*<0.001 vs Intima or Scr. HDAd: helper-dependent adenovirus; qPCR: real-time quantitative PCR.

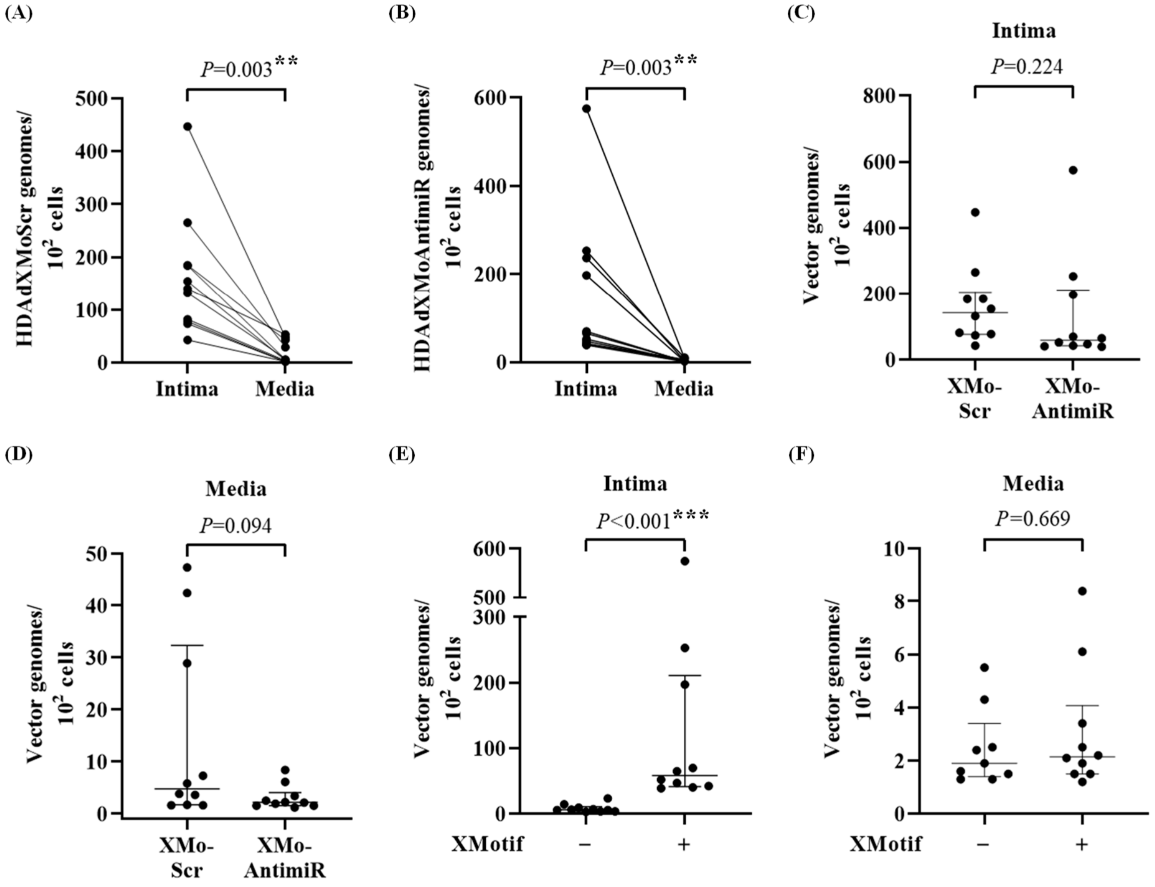

Figure S6. HDAd vector genomes in the intima and media. Rabbit carotids were treated with HDAd vectors encoding the anti-miR-33a-5p or scrambled antagomir, with or without the X-motif. Vessels were removed 3 days after treatment and intima and media were microdissected. Vector genomes in the intima and media were quantified using qPCR and normalized to the number of diploid cells (expressed as vector genomes per 100 diploid cells). (**A-B),** vector genomes in the intima and media of (**A**) HDAdXMoScr-treated and (**B**) HDAdXMoAntimiR-infused arteries. (**C-D),** vectors genomes in the (**C**) intima and (**D**) media of arteries treated with either HDAdXMoScr (XMo-Scr) or HDAdXMoAntimiR (XMo-AntimiR). (**E-F),** HDAd genomes in the (**E**) intima and (**F**) media of vessels treated with either HDAdAntimiR (–) or HDAdXMoAntimiR (+). Each data point is from the intima or media of one carotid; data points from the same carotid are connected by a line. Bars and whiskers are group medians and interquartile ranges, respectively. N=11 carotid intima and media per HDAd vector. *P* values are from (**A-B**) Wilcoxon signed-rank test and (**C-F**) Wilcoxon rank-sum test. ***P*<0.01 and ****P*<0.001 vs Intima or non-X-motif. HDAd: helper-dependent adenovirus; qPCR: real-time quantitative PCR.

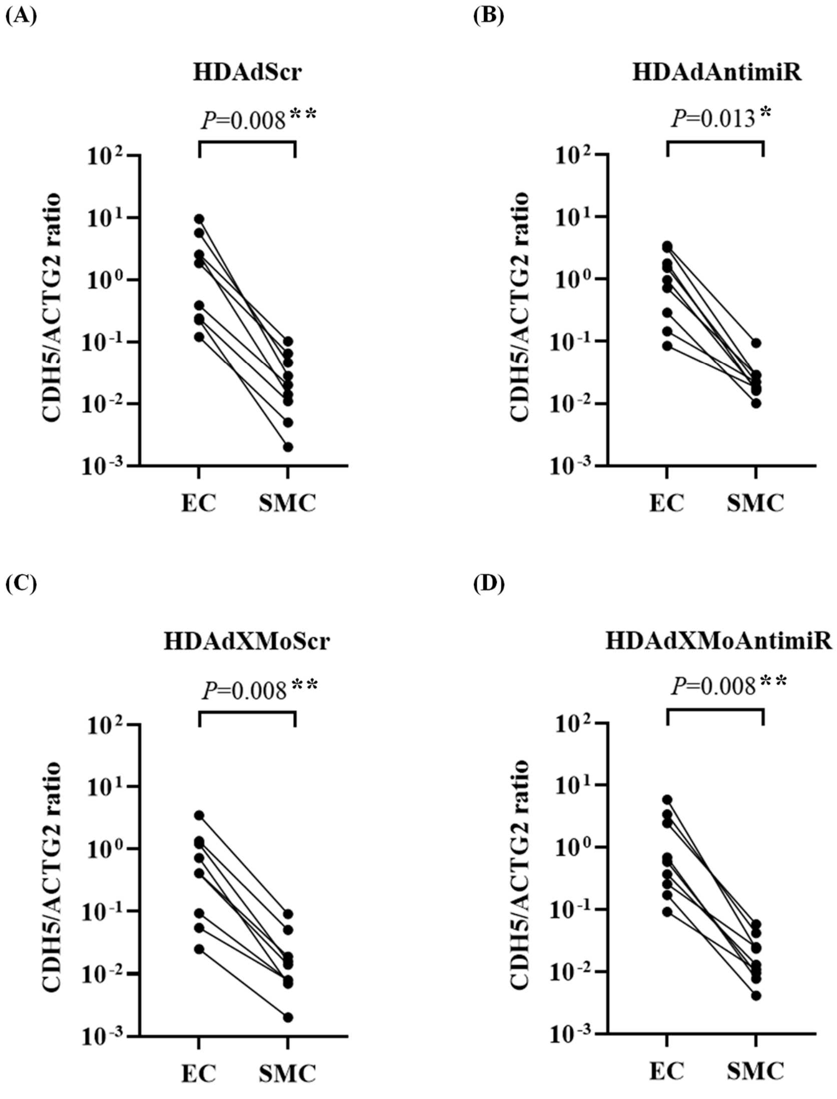

Figure S7. Cell-type markers in the sorted carotid endothelial cells (EC) and smooth muscle cells (SMC). Rabbit arteries were treated with (**A**) HDAdScr, (**B**) HDAdAntimiR, (**C**) HDAdXMoScr or (**D**) HDAdXMoAntimiR and were harvested 3 days after treatment. Vessels were enzymatically digested, and cells stained with CD45 and CD31 antibodies. The CD45-CD31+ cells (EC-enriched) and CD45-CD31- cells (SMC-enriched) were sorted for downstream RNA analysis. The cell-type markers CDH5 (enriched in endothelial cells) and ACTG2 (enriched in smooth muscle cells) were quantified in the EC and SMC extracts by qPCR. Cell-type enrichment was estimated as the ratio between CDH5 and ACTG2 expression; higher ratios indicate enrichment in EC whereas lower ratios correspond to enrichment in SMC. Each data point corresponds to the EC or SMC from one carotid; data points from the same carotid are connected by a line. N=9 carotid EC and SMC per HDAd vector. *P* values are from (**A, C-D**) Wilcoxon signed-rank test and (**B**) paired t-test. **P*<0.05 and ***P*<0.01 vs EC. HDAd: helper-dependent adenovirus; qPCR: real-time quantitative PCR.

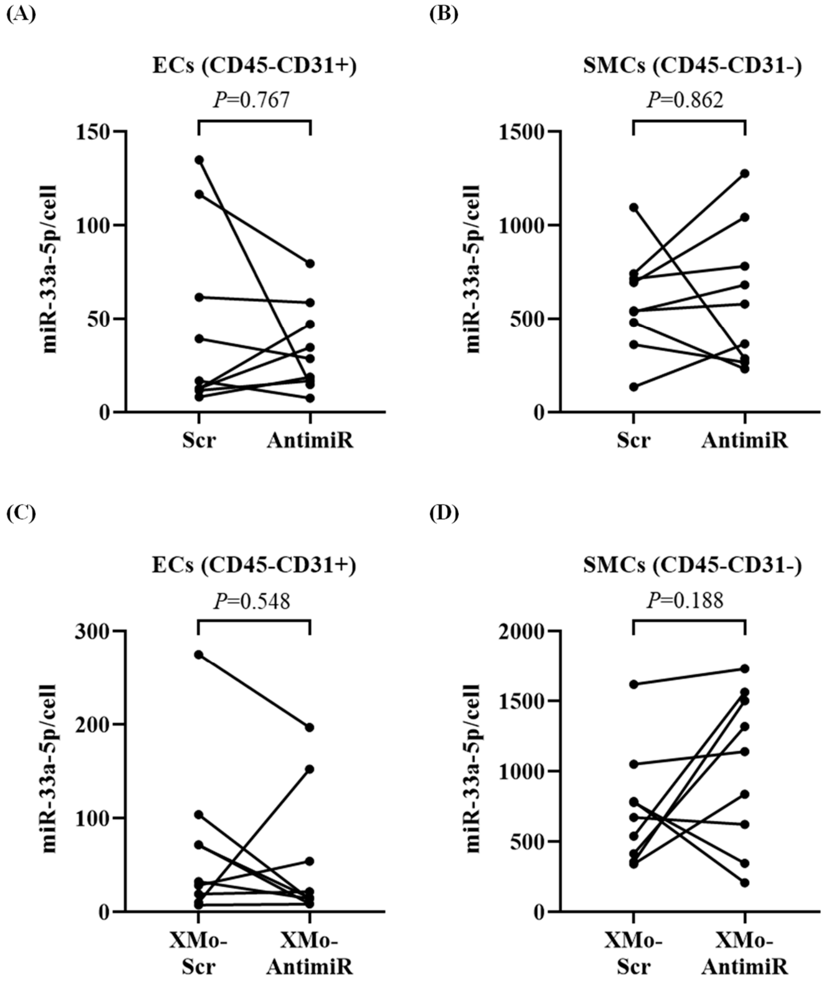

Figure S8. MiR-33a-5p in the sorted endothelial cells (EC) and smooth muscle cells (SMC). Rabbit carotids were treated with HDAd vectors encoding the anti-miR-33a-5p or a scrambled antagomir, with or without the X-motif. Arteries were removed 3 days after treatment and were enzymatically digested. Cells were stained with antibodies and EC-enriched (CD45-CD31+) and SMC-enriched (CD45-CD31-) populations were sorted for RNA analysis. The expression of miR-33a-5p was measured by qPCR, and molecule number was estimated using the standard curve (see Figure S3). Target expression was normalized to the number of sorted cells (defined as miR-33a-5p molecules per cell). (**A-B),** expression of miR-33a-5p in the (**A**) EC and (**B**) SMC of HDAdScr-infused (Scr) arteries compared to HDAdAntimiR (AntimiR) treatment. (**C-D),** expression of miR-33a-5p in the (**C**) EC and (**D**) SMC of HDAdXMoScr-treated (XMo-Scr) or HDAdXMoAntimiR-treated (XMo-AntimiR) arteries. Each data point is from the EC or SMC of one carotid; data points from the same rabbit are connected by a line. N=9 carotid EC and SMC per HDAd vector. *P* values are from (**A**) Wilcoxon signed-rank test and (**B-D**) paired t-test. HDAd: helper-dependent adenovirus; qPCR: real-time quantitative PCR.

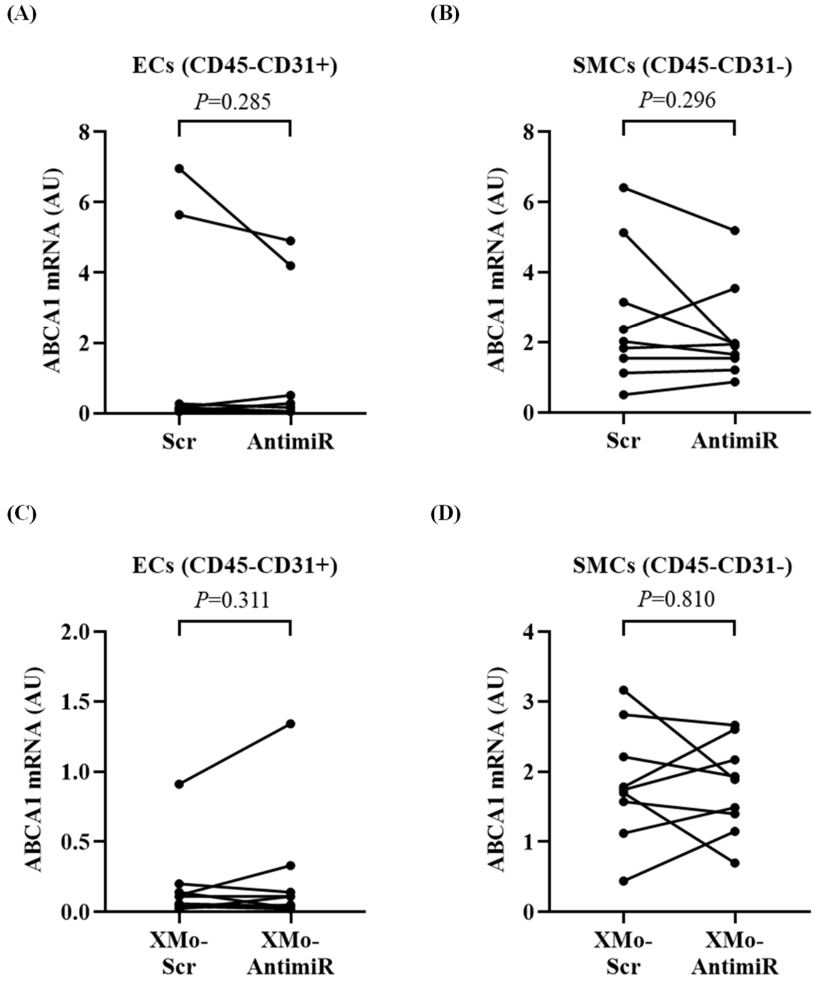

Figure S9. Expression of ABCA1 mRNA in the sorted endothelial cells (EC) and smooth muscle cells (SMC). Rabbit vessels were treated with HDAd vectors encoding either anti-miR-33a-5p or a scrambled antagomir, with or without the X-motif. After 3 days, arteries were enzymatically digested, and cells were stained with antibodies. EC-enriched (CD45-CD31+) and SMC-enriched (CD45-CD31-) populations were sorted for RNA analysis. ABCA1 mRNA expression (AU) was quantified using qPCR and normalized to the number of sorted cells. (**A-B),** expression of ABCA1 mRNA in the (**A**) EC and (**B**) SMC of HDAdScr-infused (Scr) arteries compared to HDAdAntimiR (AntimiR) treatment. (**C-D),** ABCA1 mRNA in the (**C**) EC and (**D**) SMC of HDAdXMoScr-treated (XMo-Scr) or HDAdXMoAntimiR-treated (XMo-AntimiR) arteries. Each data point is from the EC or SMC of one carotid; data points from the same rabbit are connected by a line. N=9 carotid EC and SMC per HDAd vector. *P* values are from (**A**) Wilcoxon signed-rank test and (**B-D**) paired t-test. HDAd: helper-dependent adenovirus; qPCR: real-time quantitative PCR; AU: arbitrary units.

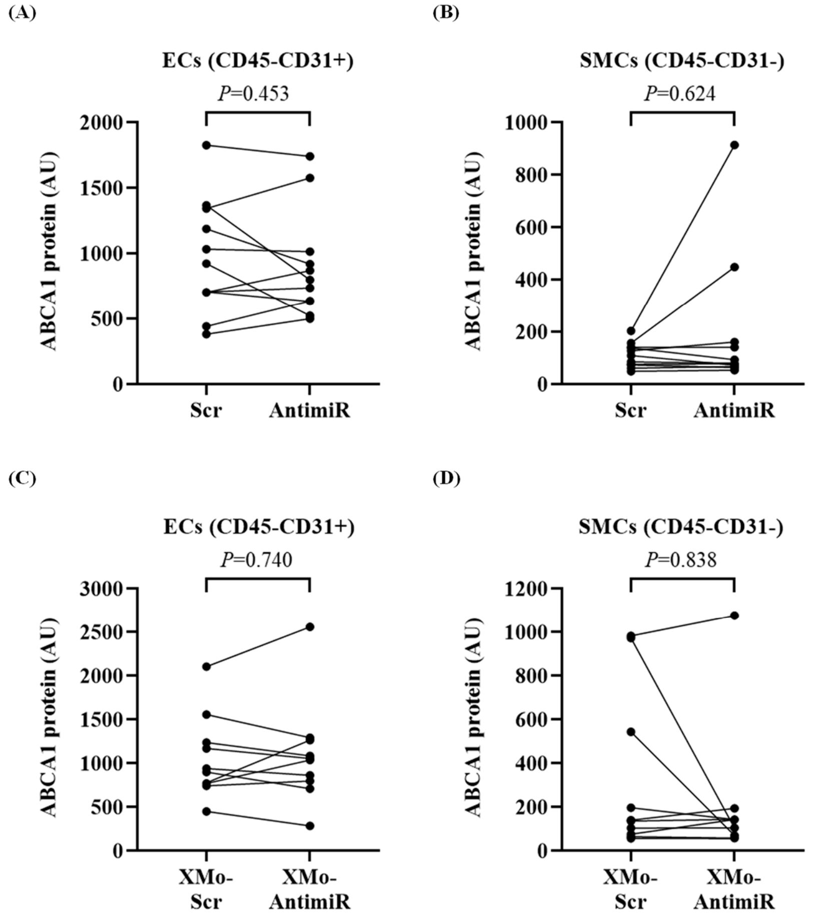

Figure S10. ABCA1 protein in the sorted endothelial cells (EC) and smooth muscle cells (SMC). Rabbit carotids were treated with HDAd vectors encoding either anti-miR-33a-5p or a scrambled antagomir, with or without the X-motif. 3 days later, vessels were enzymatically digested, and the resulting cells were stained with antibodies. ABCA1 fluorescence was measured in the EC-enriched (CD45-CD31+) and SMC-enriched (CD45-CD31-) cell populations using the cell sorter. ABCA1 protein was quantified as the median fluorescence intensity. (**A-B),** expression of ABCA1 protein in the (**A**) EC and (**B**) SMC of HDAdScr-infused (Scr) arteries compared to HDAdAntimiR (AntimiR) treatment. (**C-D),** ABCA1 protein in the (**C**) EC and (**D**) SMC of HDAdXMoScr-treated (XMo-Scr) or HDAdXMoAntimiR-treated (XMo-AntimiR) arteries. Each data point is from the EC or SMC of one carotid; data points from the same rabbit are connected by a line. N=9 carotid EC and SMC per HDAd vector. *P* values are from (**A** and **C**) paired t-test and (**B** and **D**) Wilcoxon signed-rank test. HDAd: helper-dependent adenovirus; AU: arbitrary units.

Table S1. Expression cassettes of the HDAd vectors.

Vector name	Expression cassette sequence (5′-3′)
HDAdAntimiR	GGTGCATTATAGTTGCACTGAACTTCCTGTCAGTGCAATGCAACTACAATGCACTTTTT
HDAdScr	CTAAGGTTAAGTCGCCCTCGCTCTAGCGAGGGCGACTTAACCTTAGGTTTTT
HDAdXMoAntimiR	GGTGCATTATAGTTGCACTGAACTTCCTGTCAGTGCAATGCAACTACAATGCACGGAGGAGTTTTT
HDAdXMoScr	CTAAGGTTAAGTCGCCCTCGCTCTAGCGAGGGCGACTTAACCTTAGGAGGAGTTTTT

Sequences from the transcription start site (26-nucleotides downstream from the TATA box of the H1 promoter) to the transcription termination signal (5-thymidine string). The X-motif is underlined.

Table S2. Sequences of the primers and probes.

Target name	Forward primer (5′-3′)	Reverse primer (5′-3′)	Probe (5′-3′)
E1A	AATGGCCGCCAGTCTTTTG	AAATGGCTAGGAGGTGGAAGATT	TCAGCCAGTACCTCTTCGATCAGCTGGT
HDAd (pC4HSU)	CCACCACTACATAGCCCACAGT	ACAAAGAATGGCTGAGCAAGC	TGCCCCAGCCACAGCATCCTT
Helper virus	TCTGAGTTGGCACCCCTATTC	GTTGCTGTGGTCGTTCTGGTA	TTCAGGGATGCCACATCCGTTGA
GAPDH (genomic)	CGACATCAAGAAGGTGGTGAAG	AGGGCTAAGTGTGGGAACT	TGAAGGGCATCCTGGGCTACAC
GAPDH (mRNA)	TCATTGACCTCCACTACATGGTCTA	CGCTCCTGGAAGATGGTGAT	None
CDH5	AGGACACAACGCCACAAA	TCAAACTGCCCGTACTTGAC	None
ACTG2	TGCTGACAGGATGCAGAAG	CCCGATCCAGACCGAGTA	None
ABCA1	AGTCTGTGTTCTTGATCTTCCC	TTCTCCCCAAACCTTTCCAG	None

Sequences of the primers and probes (IDT) used for the real-time quantitative PCR. Target genes without probe were measured using SYBR Green reagent (ABclonal).

Table S3. Target sequences for the quantitative PCR of small RNA.

Target name	Target sequence (5′-3′)	Cat. number (QIAGEN)
Anti-miR-33a-5p	UGCAAUGCAACUACAAUGCACUUUU	YCP2158550
XMo-anti-miR-33a-5p	UGCAAUGCAACUACAAUGCACGGAGGAGUUUU	YCP2158552
miR-33a-5p	GUGCAUUGUAGUUGCAUUGCA	YP00205690
snU6	Not provided by the manufacturer	YP02119464

Target sequences and catalog number of the primers used for the quantitative PCR of small RNAs. Primers for anti-miR-33a-5p and XMo-anti-miR-33a-5p were custom made.
